# Supplementary material for: Inflammatory Surrogate Parameters for Predicting Ifosfamide-Induced Neurotoxicity in Sarcoma Patients
Source: J Clin Med. 2022 Sep 30;11(19):5798. doi: 10.3390/jcm11195798 (PMC9572151; doi:10.3390/jcm11195798)
Supplement: Supplementary file 1 [file jcm-11-05798-s001.zip › jcm-1907278-supplementary.pdf]

Journal of
Clinical Medicine

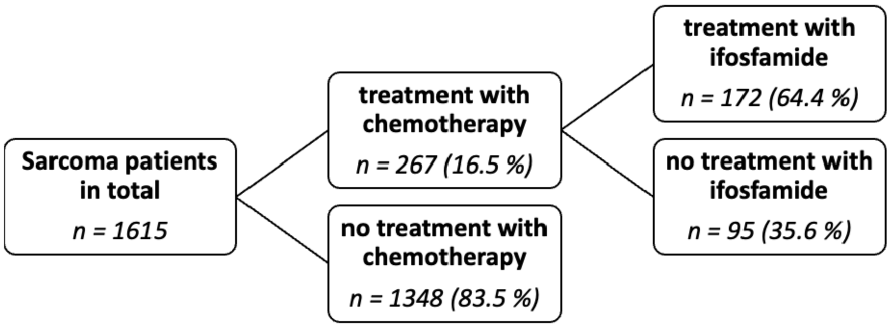

Supplementary Figure S1. Flow chart describing patient selection

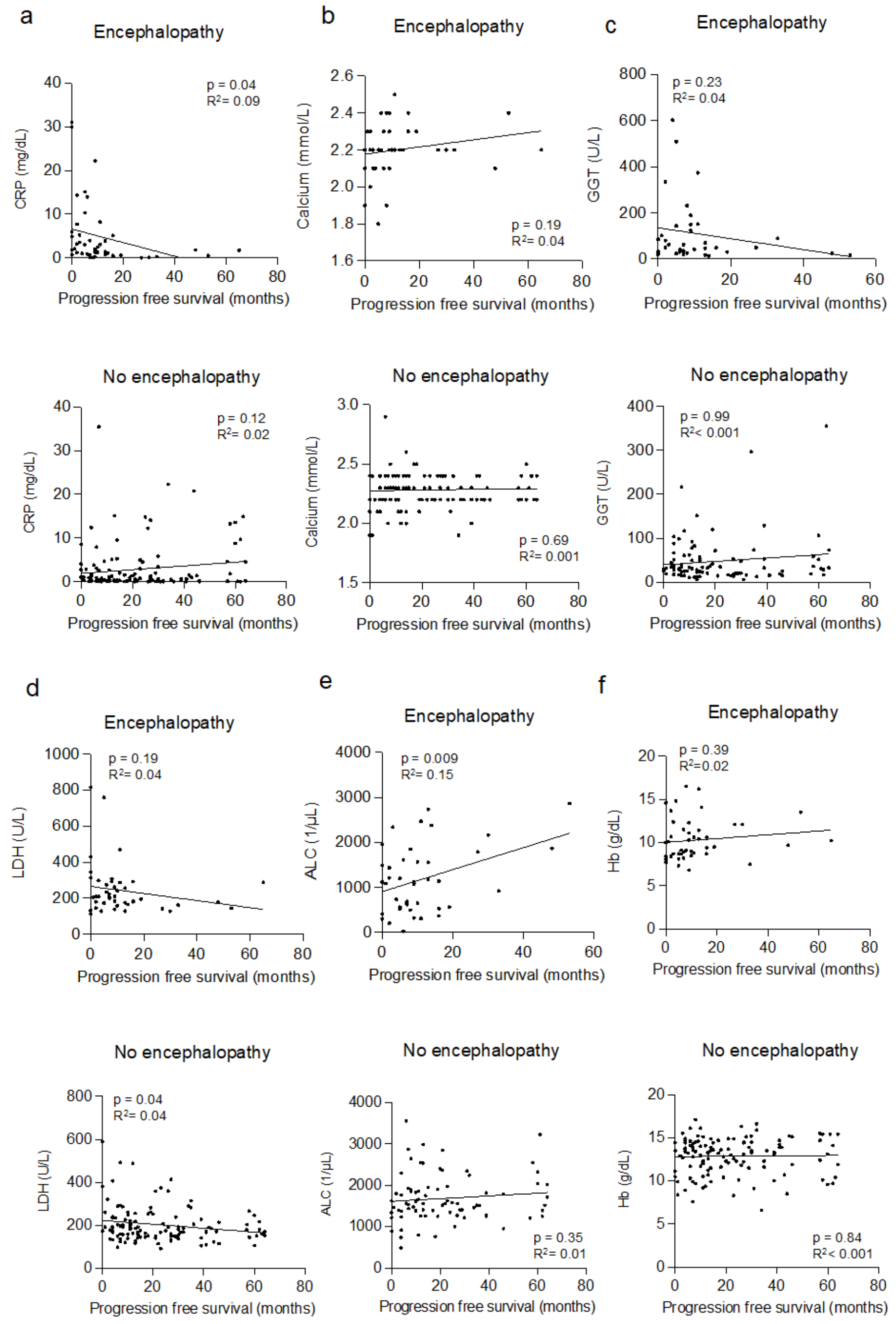

Supplementary Figure S2. Association of CRP (a), calcium (b), GGT (c), LDH (d), ALC (e) and HB level (f) and PFS. CRP = C-reactive protein, GGT = gamma-glutamyl transferase, ALC = Absolute lymphocyte count, Hb = Hemoglobin level, LDH = Lactate dehydrogenase

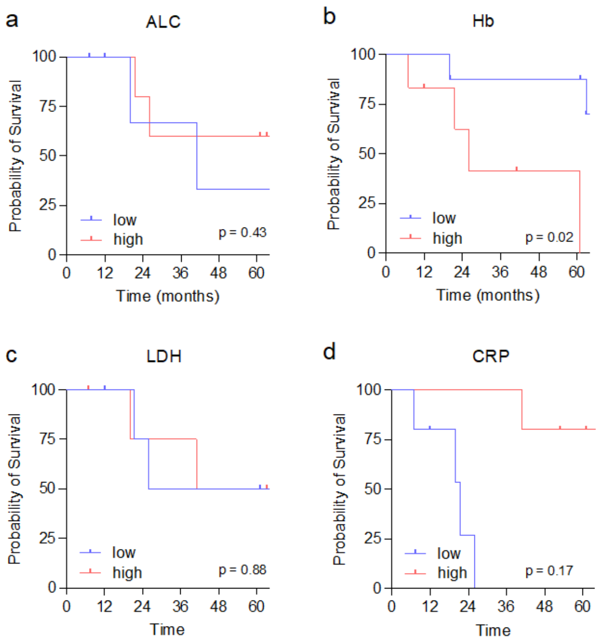

Supplementary Figure S3. Kaplan-Meier curves showing the Influence of ALC (a), Hb (b), GGT (c), LDH and CRP level (d) in patients without ifosfamide treatment and probability of survival. ALC = Absolute lym-phocyte count.

Supplementary Table S1: Occurrence of IIN in sarcoma patients

**Median follow-up (months)**	IIN	without IIN
Time to progression	8	17
Time to death	16	27
**Mean follow-up (months)**		
Time to progression	11.00	22.05
Time to death	22.28	28.41
**Duration of IIN**		
Mean duration of IIN (days)	3.17	
Median duration of IIN (days)	3.00	
**Occurrence of IIN**		
Mean time until occurrence of IIN (hours)	43.97	
Median time until occurrence of IIN (hours)	40.00	

	n	%
< 24 hours until occurrence of IIN	9	24.32
24 - 48 hours until occurrence of IIN	15	40.54
49 - 72 hours until occurrence of IIN	9	24.32
> 72 hours until occurrence of IIN	4	10.81
**Treatment of IIN**		
	n	%
Administration of thiamine	29	78.38
Administration of methylene blue	22	59.46
Administration of haloperidol	4	10.81

Supplementary Table S2: Patient characteristics in sarcoma patients without ifosfamide

Patient characteristics	Total	(%)
	(n=10)	
**Gender**		
female, sex (n)	63	36.6
**Age**	29 to 84	
Age in years, mean–yr. ± SD	53.0 +/- 16.2	
**TNM classification**		
Stage		
Tx	0	0
T1	0	0
T2	8	80
T3	1	10
T4	1	10
Node		
Nx	1	10
N0	5	50
N1	4	40
N2	0	0
N3	0	0
Metastasis		
Mx	0	0
M0	5	50
M1	5	0
**Histology**		

Leiomyosarcoma	3	30
Liposarcoma	2	20
Myxofibrosarcoma	2	20
Undifferentiated pleomorphic sarcoma	3	30
**UICC stage**		
X	0	0
I	3	30
II	0	0
III	5	50
IV	2	20
**Treatment**		
Doxorubicin	4	40
Gemcitabine	2	20
Pazopanib	1	10
Olaratumab	3	30
